# Antibiofilm and Immune Response of Engineered Bioactive Nanoparticles for Endodontic Disinfection

**DOI:** 10.3390/jcm9030730

**Published:** 2020-03-09

**Authors:** Hebatullah Hussein, Anil Kishen

**Affiliations:** 1 The Kishen Lab, Dental Research Institute, University of Toronto, Toronto, ON M5G 1G6, Canada; hebatullah.hussein@mail.utoronto.ca; 2 Faculty of Dentistry, University of Toronto, Toronto, ON M5G 1G6, Canada; 3 School of Graduate Studies, University of Toronto, Toronto, ON M5G 1G6, Canada; 4 Department of Dentistry, Mount Sinai Health System, Mount Sinai Hospital, Toronto, ON M5G 1X5, Canada

**Keywords:** engineered chitosan nanoparticles, root canal disinfection, *Enterococcus faecalis* biofilm, RAW macrophages, inflammation

## Abstract

The biological aim of root canal treatment is to facilitate periapical tissue healing following endodontic therapy. This study aimed to develop an organotypic infected root canal model to understand the interaction of bacterial biofilm with macrophages and study the therapeutic effect of engineered bioactive chitosan nanoparticles (CSnp) on macrophages. Ex-vivo experiments were conducted in two phases; Phase-1: *Enterococcus faecalis* biofilms (two and six weeks old) developed in organotypic root canal model were used to characterize residual biofilm after conventional chemical treatment alone and combined with CSnp utilizing Confocal Laser Scanning Microscopy, Scanning Electron Microscopy and colony-forming units from pulverized dentin. Phase-2: The interaction of post-treatment biofilm and RAW macrophages was evaluated regarding pro/anti-inflammatory markers, cell viability and spreading at 24, 48 and 72 h. Compared to conventionally disinfected six-week-old biofilm, CSnp resulted in less viable bacteria (*p* < 0.01). Scanning electron micrographs demonstrated disruption of the biofilm. CSnp exhibited less residual bacterial load in pulverized dentin (*p* < 0.001). Macrophage interaction with CSnp-treated biofilm reduced proinflammatory markers (nitric oxide, TNF-α, IL-1β, and IL-6), increased anti-inflammatory marker (TGF-β1) and enhanced cell survival and spreading over time (*p* < 0.01 at 72 h). Engineered chitosan nanoparticles concurrently inactivated biofilm and altered the inflammatory response of macrophages that would promote healing.

## 1. Introduction

Healing of the periapical tissue is the ultimate biological aim of root canal treatment [1]. Apical periodontitis is an inflammatory disorder of periradicular tissues caused by etiological agents of endodontic origin [2,3]. It is caused primarily by bacteria organized as biofilm within the root canal system [4]. Thus, its treatment requires disinfection of infected root canal dentin, minimization of bacterial persistence and promotion of post treatment healing [5]. Despite technological advancements in endodontics, several studies have shown that total elimination of bacterial biofilm from the root canal system could not be achieved. Residual microbial biofilms and their concomitant interaction with the host immune system is crucial for the development of persistent [6] or chronic inflammatory reaction that ultimately contribute to post treatment failure [7]. 

A substantial portion of the periradicular tissue damage that characterizes apical periodontitis can be attributed to the host immune response to the presence of intracanal bacteria [7]. Macrophages are crucial modulators in the regulation of inflammation, tissue repair and regeneration of periradicular tissues. They could be polarized into classically activated M1 cells (proinflammatory) and alternatively activated M2 cells (anti-inflammatory/healing), depending on their exposure to different stimuli [8]. Several cytokines secreted by macrophages are involved in the pathogenesis and progression of apical periodontitis [9,10]. Modulation of the host immune response to infection could be achieved by application of medications capable of controlling the inflammatory response. However, there is no current therapeutic approach applied in endodontic treatment that could target periapical inflammatory response to maximize favorable conditions for healing. 

Chemical disinfectants are indispensable during root canal treatment. For enhanced control of infection, several nanomaterials have been applied as root canal irrigants such as; metal-based, polymeric, bioactive glass and calcium derivatives nanoparticles, and intracanal medications as well, such as; silver, zinc oxide and chitosan nanoparticles, aiming at good biocompatibility and improving the antimicrobial activity [11,12]. Engineered bioactive chitosan nanoparticles (CSnp) have been shown to effectively inactivate bacterial biofilm and disrupt its extracellular polymeric matrix [13,14]. They have been reported to possess an increased affinity to bacterial cell membrane, higher penetration into biofilm structure [13], as well as eliminate bacterial mono-species and clinically relevant multispecies biofilm on a time-dependent interaction [14] and thus present a potential antimicrobial/antibiofilm agent for root canal disinfection [15,16]. Carboxymethyl chitosan (CMCS), a water-soluble derivative of chitosan, is biodegradable, biocompatible, nontoxic antibacterial polymer [17], that has been reported as a surface modifier of dentin matrix to enhance antibacterial efficacy [13,18]. Engineered chitosan-based nanoparticles as a bioactive biopolymer capable of interacting with eukaryotic cells might alter the response of immune host cells to intracanal infection. We hypothesize that engineered bioactive chitosan nanoparticles inactivate bacterial biofilm and alter host inflammatory response of macrophages to promote healing. 

The purpose of this study is to investigate the ability of engineered bioactive chitosan nanoparticles as a medication to disinfect root canal biofilm and modulate inflammatory response of macrophages in endodontic treatment.

## 2. Materials and Methods

**Figure 1:**
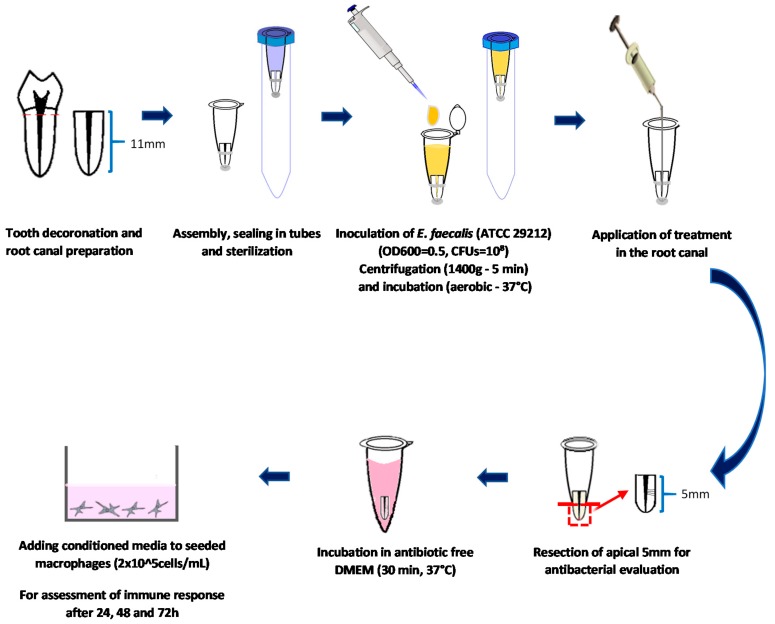
Schematic representation of the study methodology.

All the chemicals used in this study were of analytic grade (purity ≥ 95%) and were purchased from Sigma-Aldrich Inc (St. Louis, MO, USA) unless otherwise stated. Engineered chitosan nanoparticles previously synthesized and characterized in the Kishen lab [14,19] were used. This study was approved by the Ethics Review Board at the University of Toronto (protocol reference #35228, original approval date: 1 December 2017). Figure 1 depicts a schematic representation of the methodology. 

### 2.1. Phase-1: Characterization of Post-Disinfection Biofilm Model

#### 2.1.1. Teeth Selection and Preparation

Single-rooted, single canal human extracted teeth were decoronated using rotary wheel saw to achieve a length of 11 mm. The presence of single canals was confirmed through buccolingual and mesiodistal radiographs and a total of 128 teeth were included in the study. ProTaper Universal rotary instruments (Dentsply Tulsa Dental Specialties, Tulsa, OK, USA) were used to sequentially enlarge root canals at 10 mm working length up to F3 at 300 rpm and 200 g/cm torque (ProMark Endodontic Motor, Dentsply Sirona) and apical patency was performed throughout instrumentation using ISO size 10 K-files. For canals irrigation, 3 mL of 3% sodium hypochlorite (NaOCl, purchased from Lavo Inc (Montreal, Quebec, Canada)) delivered at each change of file during instrumentation, 1mL of 17% ethylene diamine tetra-acetic acid (EDTA) for smear layer removal and final flush with sterile deionized water (DIW) were used. The apical 7 mm of each root was extruded from the bottom of 1.5 mL eppendorf tube and coated with epoxy resin to seal the outer root surface and the apical foramen. Samples were assembled individually in 15 mL tubes and autoclaved for sterilization at 121 °C for 20 min. All subsequent procedures were carried out in a biosafety cabinet using sterile instruments to ensure sterility of the procedures.

#### 2.1.2. Mono-Species Biofilm Formation

A suspension obtained from an overnight incubated culture of *E. faecalis* (ATCC 29212, American Type Culture Collection, Manassas, VA, USA) in sterile brain heart infusion (BHI) broth (OD_600nm_ = 1, colony-forming units (CFUs) = 10^8^) was used. Each root canal (n = 24/group) was filled to the orifice level and 1mL of bacterial suspension was added to the tube. Samples were centrifuged at 1400× *g* for 5 min to facilitate bacterial penetration into dentinal tubules [20] and incubated at 37 °C for 2 and 6 weeks with the medium being changed every 48 h. Negative control specimens (n = 8) were not inoculated to check for the sterility of the procedures. 

#### 2.1.3. Disinfection Procedures 

Engineered CSnp was applied following conventional disinfection of 2 and 6-week-old root canal biofilms. The root canals (n = 12/group at each biofilm age) were treated using the following protocols: 

Group 1 (Untreated): Positive control to provide baseline for biofilm characterization. 

Group 2 (Conventional chemicals): Each canal was irrigated using 3 mL of 6% NaOCl delivered with 27-gauge needle inserted 1mm short of the working length, followed by 3 mL of 5% sodium thiosulphate to inactivate NaOCl, then 2 mL 17% EDTA for 1 min and final flush with sterile saline solution. 

Group 3 (CSnp/DIW): Root canals were treated as in group 2, followed by application of engineered CSnp dispersed in sterile deionized water (10 mg/mL) for 72 h. 

Group 4 (CSnp/CMCS): was treated as in group 2 as well, followed by the application of CSnp dispersed in 1% CMCS solution (1 mg/mL) for 72 h. 

#### 2.1.4. Evaluation of Antimicrobial Efficacy

The apical 5 mm of the treated roots were resected horizontally using rotary wheel saw and prepared for the evaluation of disinfection efficacy using the following methods: 

Confocal Laser Scanning Microscopy: Quorum Spinning Disk Confocal microscope (Olympus IX81) was used to study the bacterial killing ability in the dentinal tubules and on the surface of root canal lumen. Longitudinal apical root sections were stained using LIVE/DEAD BackLight Bacterial Viability Kit (Molecular Probes, Inc, Eugene, OR, USA) for 15 min (SYTO 9 stain: excitation/emission = 480/500 nm, Propidium iodide stain: excitation/emission = 490/635 nm). Images were acquired at 10× and 40× magnification. Perkin Elmer Volocity software was used to calculate the volume (μm^3^) of the live and dead bacteria. 

Scanning Electron Microscopy: QUANTA FEG 250 ESEM was used to characterize the biofilm uniformity, thickness, abundance of extracellular polymeric substance and confirm bacterial penetration into dentinal tubules. Apical root sections were fixed in 2.5% glutaraldehyde at 4 °C overnight, dehydrated in graded series of ethanol, split longitudinally, followed by critical point drying and finally sputter coated with platinum 2 nm in thickness. Qualitative description based on the micrographs was done without further quantitative analysis because the extensive sample preparation steps can affect the original biofilm morphology [21], besides it cannot differentiate live from dead bacterial cells. 

Culture analysis of pulverized dentin: A freezer mill (6755, Spex, Metuchen, NJ, USA) operated at liquid nitrogen temperature was used to cryogenically grind apical root sections (*n* = 6/group). Each specimen was weighed then crushed and the pulverized dentin was suspended in 1mL of sterile BHI broth and agitated in vortex for 1 min. Following bacterial enrichment for 3 h at 37 °C, ten-fold serial dilutions were done and 10 μL aliquots were plated onto BHI agar (in triplicates) and incubated at 37 °C for 48 h. The CFUs were enumerated and using each specimen’s weight, CFUs/mg was calculated. 

### 2.2. Phase-2: Interaction of Engineered Nanoparticles Treated Biofilm and Macrophages 

#### 2.2.1. Cell Culture

RAW264.7 (ATCC TIB-71) macrophages of third to fifth passage were grown to 85% confluency in Dulbecco’s Modified Eagle’s medium (DMEM) supplemented with 10% heat inactivated fetal bovine serum (FBS), 1% Penicillin/Streptomycin in humidified incubator at 37 °C, 5% CO_2_. Macrophages were detached using rubber cell scraper, seeded in multi-well plates at cells concentration of 2 × 10^5^/mL and incubated overnight at 37 °C, 5% CO_2_ for cells attachment.

#### 2.2.2. Interaction of Treated Biofilm and Macrophages Cell Culture

Root canal models with 6-week-old *E. faecalis* biofilm, treated following the same disinfection protocols described in phase 1 (*n* = 6/group), were utilized to assess the interaction between residual biofilm and RAW macrophages. The apical 5 mm of each root sample was resected horizontally, its outer surface was disinfected using 70% ethanol and sterility of the external root surface was checked by sampling from the outer root surface and incubation overnight. Each specimen was incubated in 2 mL of antibiotic free cell culture media (DMEM + 10% FBS) at 37 °C for 30 min. The conditioned media was centrifuged twice at 3000× *g* for 5 min, added at 2-fold dilution to the overnight cultured macrophages and incubated in humidified incubator at 37 °C, 5% CO_2_ for assessment in triplicates after 24, 48 and 72 h of interaction. 

Cell viability assessment: Direct staining with calcein AM (Invitrogen/Molecular Probes) stain was performed to assess cell viability over time. Cells were incubated for 20 min with 200 μL calcein AM then observed using a fluorescent microscope (Vert.A1; Carl Zeiss, Jena, Germany) at 10 and 20× magnification. ImageJ software (U.S. National Institutes of Health, Bethesda, MD, USA) was used for images analysis to determine the viable cell counts in microscopic fields. Cell viability was expressed as percentage survival normalized to unstimulated macrophages. Trypan blue exclusion assay was done to confirm the cells viability.

Nitric oxide release: Griess reagent system (Promega, Madison, WI, USA) was used to evaluate nitrite (NO_2_)^−^ released in the cell culture supernatant collected, according to the manufacturer’s instructions. The nitric oxide (NO) concentration was determined quantitatively using the standard curve of nitrite [16].

Production of inflammatory mediators: The concentrations of tumor necrosis factor alpha (TNF-α), interleukin 1 beta (IL-1β), interleukin 6 (IL-6), and transforming growth factor beta 1 (TGF-β1) in RAW264.7 cell culture supernatants were analyzed using commercial enzyme-linked immunosorbent assay kits (Quantikine ELISA, Mouse TNF-α, IL-1β, IL-6 and TGF-β1 Immunoassays; R&D, Minneapolis, MN, USA). For detection of IL-1β cytokine, 5 mM ATP was added in separate wells for 30 min prior to collection of cell culture supernatant to activate RAW cells to produce the active form of IL-1β. Each assay was performed according to the manufacturer’s instructions. Purified recombinant TNF- α, IL-1β, IL-6 and TGF-β1 were used as the standards. The results were expressed in pg/mL read off the standard curve. 

Cells spreading assessment: Microscopic images of calcein AM stained cells were used to calculate the spreading area of macrophages in regions of interest using ImageJ software (U.S. National Institutes of Health, Bethesda, MD, USA).

### 2.3. Statistical Analysis

Data obtained were expressed as the mean and standard error of the mean. Statistical analyses were performed using one-way analysis of variance and post hoc Tukey test in case of significance. Kruskal–Wallis and Dunn tests were used to perform multiple comparisons of the biofilm volumes obtained from CLSM. A *p*-value < 0.05 was considered statistically significant.

## 3. Results 

### 3.1. Phase-1: Characterization of Post-Disinfection Biofilm Model

**Figure 2:**
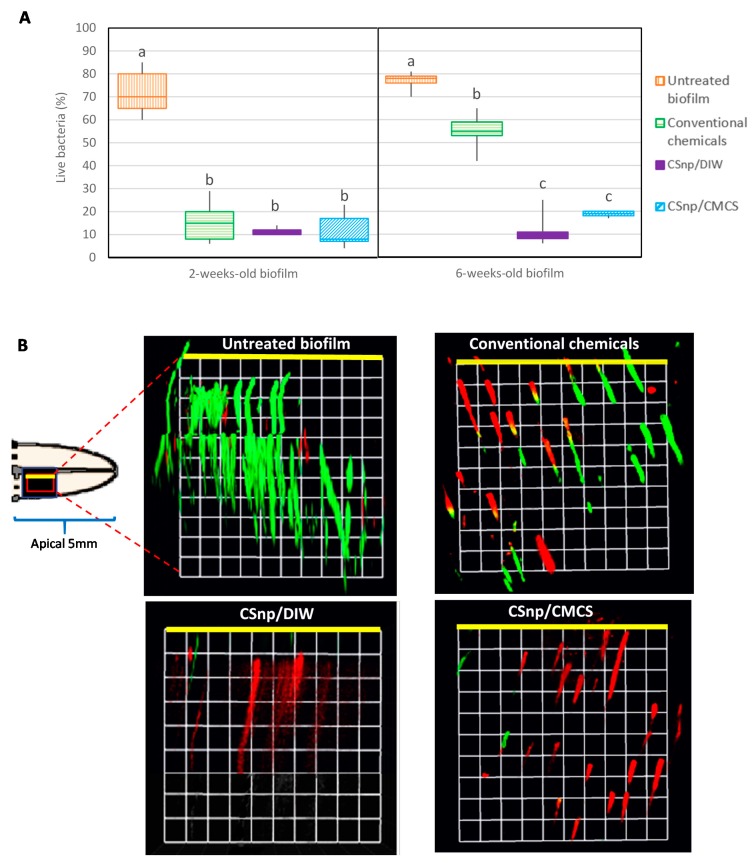
(**A**) Confocal laser scanning microscopy evaluation of antibacterial efficacy of CSnp/CMCS and CSnp/DIW versus conventional chemicals on 2- and 6-week-old *E. faecalis* biofilm showing percentage of live bacteria. Different characters above bars indicate statistically significant difference between the groups (*p* < 0.01). (**B**) Confocal images of live/dead stained bacteria in root canal dentinal tubules of untreated and treated 6-week-old *E. faecalis* biofilm (green color: live bacteria, red color: dead bacteria, 40× magnification, 1 unit = 11.52 μm), the yellow line represents the interface between root canal lumen and the dentinal tubules in root canal wall.

Confocal images analyses revealed that the percentages of bacteria surviving on the root canal dentin and within the dentinal tubules in group 1 (untreated) were 72% and 76.8% in 2- and 6-week-old biofilms respectively. In group 2, conventional chemicals reduced the viable bacteria to 15.78% in the 2-week-old biofilm, whereas about 54.7% live bacteria survived in the 6-week-old biofilm. CSnp/DIW and CSnp/CMCS resulted in higher disruption of biofilm-structure and bacterial killing of 6-week-old biofilms compared to group 2 (*p* < 0.01) (Figure 2). 

**Figure 3:**
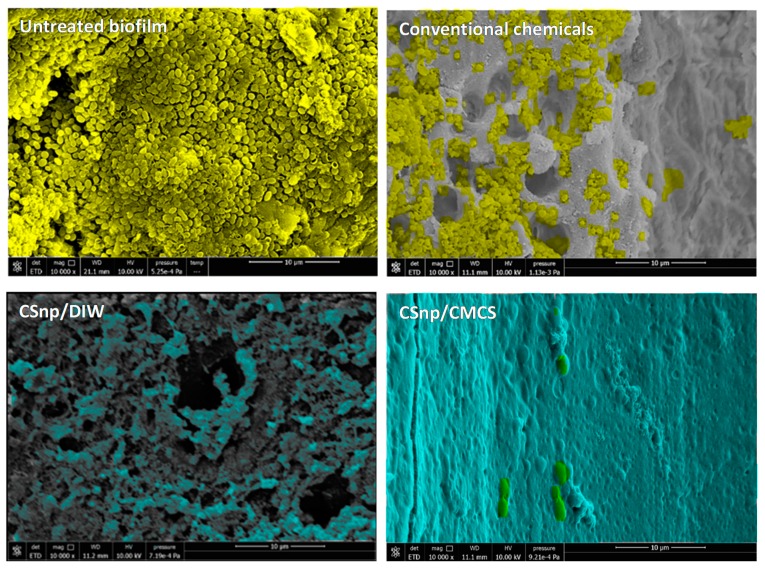
Scanning electron micrographs showing representative areas of root canal lumen of different groups of 6-week-old *E. faecalis* biofilm. Untreated biofilm displaying thick bacterial layer on the root canal wall and conventionally treated biofilm showing residual bacteria (pseudo-colored in yellow) versus CSnp treated biofilms which show CSnp-based layer covering the dentin surface (pseudo-colored in turquoise) (10,000× magnification).

Scanning electron micrographs demonstrated that in group 1, *E. faecalis* consistently colonized root canal dentin surfaces, invaded towards the dentinal tubules and formed a biofilm after 2 weeks of incubation with detected extracellular polymeric substance. After 6 weeks, a thick multi-layered biofilm with abundant extracellular polymeric substance was formed with heavy penetration into dentinal tubules. In group 2, upon conventional disinfection 2-week-old biofilm was mostly eliminated from previously colonized dentin surfaces and dentinal tubules with some residual bacteria detected. However, in 6-week-old biofilm conventional disinfection resulted in uneven disruption of the biofilm with regions of residual biofilm on root canal walls and areas of open dentinal tubules. In groups 3 and 4, CSnp treated root canal biofilm, fewer residual bacterial cells were evident and a CSnp based layer was identified covering the root canal dentin (Figure 3).

**Figure 4:**
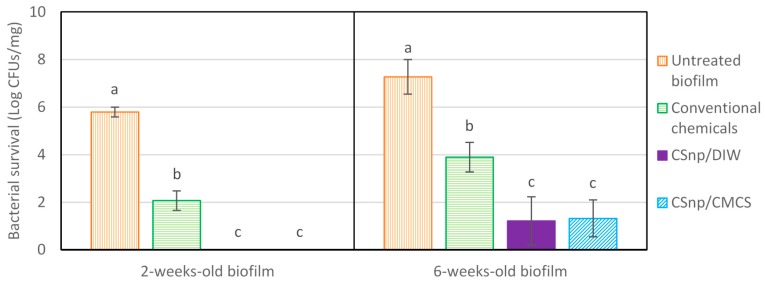
Microbiological evaluation of antibacterial efficacy of CSnp formulations versus conventional chemicals on 2- and 6-week-old *E. faecalis* biofilm aliquoted following pulverization and bacterial enrichment. Different characters above bars indicate statistically significant difference between the groups at each biofilm age (*p* < 0.001).

Microbiological quantification of pulverized dentin showed that in group 1 (untreated), 2- and 6-week old biofilms demonstrated (5.79 ± 0.21) and (7.27 ± 0.71) bacterial log respectively. Group 2, conventionally disinfected 2-week-old biofilm resulted in 3.7 log reduction of the initial bacterial load (2.07 ± 0.41 log residual bacteria) (*p* < 0.001), (Figure 4). However, the 6-week-old biofilm showed more resistance to bacterial killing with 3.37 log reductions after conventional disinfection (3.9 ± 0.62 log residual bacterial load). Groups 3 and 4 (CSnp treated 2-week-old biofilm) showed no bacterial growth. Groups 3 and 4 treated 6-week-old biofilms resulted in (1.21 ± 1.01) and (1.32 ± 0.78) logs of residual bacterial loads respectively. Both groups of engineered nanoparticles treated 2- and 6-week-old biofilms resulted in significantly less viable bacteria than conventional disinfection (*p* < 0.001), with no significant difference between group 3 (CSnp/DIW) and group 4 (CSnp/CMCS) (*p* > 0.05). Negative control samples resulted in no bacterial growth.

### 3.2. Phase 2: Interaction of Nanoparticles Treated Biofilm and Macrophages 

**Figure 5:**
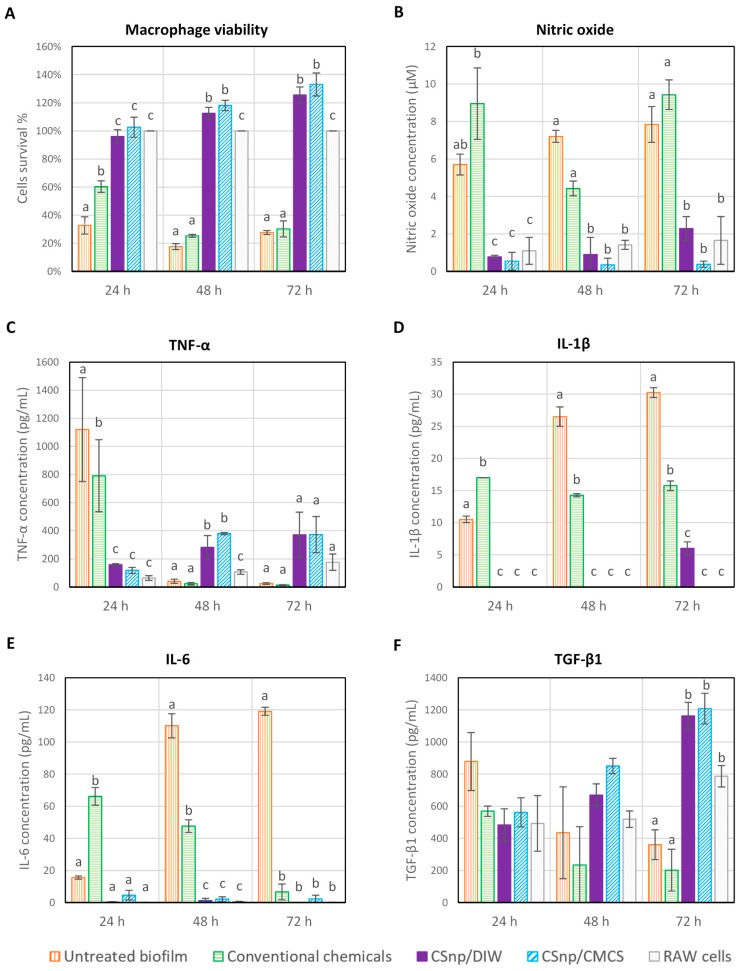
Macrophage survival and inflammatory markers production following interaction of treated 6-week-old *E. faecalis* biofilm and RAW264.7 macrophages (**A**) Relative cell viability, (**B**) Nitric oxide release, (**C**) TNF-α level (**D**) IL-1β level, (**E**) IL-6 level and (**F**) TGF-β1 level. Error bars represent standard error of the mean, different characters above bars indicate statistically significant difference between the groups at each time point (*p* < 0.01).

**Figure 6:**
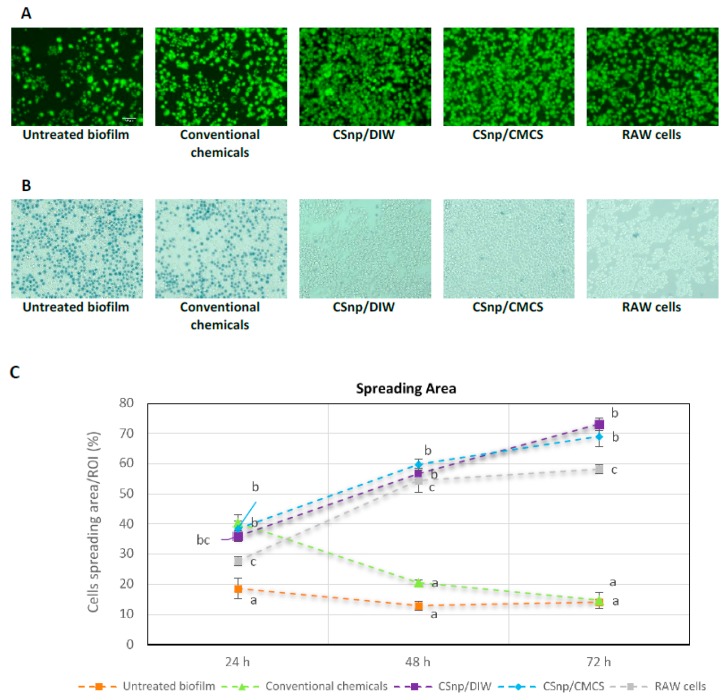
(**A**) Fluorescent images of Calcein AM stained RAW264.7 macrophages showing relative viability of cells after 72 h of interaction, (20× magnification, scale bar = 50 μm). (**B**) Microscopic images showing trypan blue exclusion assay performed to check cells viability in different treatment groups. (**C**) Spreading area of live macrophages in regions of interest (ROI) in treatment groups showing highest percentage of spreading in both CSnp treated groups. Error bars represent standard error of the mean, different characters above bars indicate statistically significant difference between the groups at each time point (*p* < 0.01).

In group 1, upon interaction of untreated biofilm and RAW264.7 macrophages, cell viability reduced significantly overtime. Similarly, in group 2, conventional disinfection reduced cells viability after 48 and 72 h of interaction. Conversely, the relative cells viability increased over time in both groups 3 and 4 (CSnp treated biofilm) (Figure 5A and Figure 6A,B). Compared to untreated and conventionally treated biofilm, CSnp resulted in significant reduction in nitric oxide released (*p* < 0.01). Proinflammatory cytokine (TNF-α) production by RAW macrophages stimulated with untreated biofilm peaked at 24 h and then dropped distinctly at 48 and 72 h of interaction. In comparison to group 2 (conventional chemicals), CSnp in groups 3 and 4 resulted in more suppression of TNF-α level at 24 h. Groups 3 and 4 (CSnp/DIW and CSnp/CMCS) treated biofilms showed higher TNF-α level compared to negative control at 48 h, then insignificant differences were found at 72 h. Group 1 (untreated) and 2 (conventional chemicals) resulted in significantly higher IL-1β and IL-6 cytokines production at 24 and 48 h of interaction (*p* < 0.01). IL-1β was detected in CSnp treated group only at 72 h at significantly lower level than conventional treatment. After 48 and 72 h, interaction between RAW cells and CSnp treated biofilms resulted in increased TGF-β1 production when compared to its level in conventionally treated biofilm group (Figure 5). At similar timepoints, highest percentage of macrophages spreading area was observed in both CSnp treated groups (*p* < 0.01), (Figure 6C).

## 4. Discussion

Microbial sampling of endodontically treated teeth with persistent or secondary periapical disease have been shown to be dominated by gram-positive facultative bacteria such as enterococci [22]. *Enterococcus faecalis* biofilm was used as a model organism in this study as its ability to form biofilm, virulence factors and survival under harsh environmental conditions, such as post-treatment endodontic conditions, play a critical role in its association to failure of root canal treatment [23,24]. Further, *E. faecalis* is resistant to the antimicrobial effects of the most commonly used intracanal medication, calcium hydroxide, possibly due to an effective proton pump mechanism, which preserves optimal cytoplasmic pH levels [25]. Although *E. faecalis* has been widely utilized and investigated in endodontic microbiology research [13,20], limited information is available about the inflammatory response of macrophages to post-treatment residual biofilm. Moreover, the effect of engineered bioactive CSnp, as a novel therapeutic approach for endodontic disinfection, on such interaction between residual biofilm and macrophages has not been investigated. 

Calcium hydroxide has been used extensively in endodontic disinfection models as an intracanal medication; however, it was not able to eliminate microbes even after prolonged exposure. *E. faecalis* can grow at high pH up to 11.1 enabling it to resist the bactericidal effect of calcium hydroxide, which eventually provides ecological selectivity for persistent infection by *E. faecalis*. Moreover, *E. faecalis* has the capacity to form distinct calcified biofilm in a calcium carbonate and calcium phosphate rich microenvironment, which could be one of the contributing factors that allow the persistence of *E. faecalis* in endodontically treated teeth [26]. Calcium hydroxide could require 7–10 days of intracanal application to exert its antibacterial action [27]. Complete elimination of calcium hydroxide prior to obturation using conventional methods remains a challenge especially in the apical portion of root canals [28,29] with the possibility of transportation in curved canals during retrieval [30]. Furthermore, calcium hydroxide has been shown to possess cytotoxic effects on macrophages [16] with no proven ability to modulate the periapical tissue inflammation, thus, it was not included in this study as a control group.

CSnp-based medications were applied intracanal for 72 h to allow for enough contact time required for elimination of biofilm. The negatively charged biofilm extracellular polymeric substance might resist the penetration of CSnp and also serve as a chemical barrier by adsorbing the harmful reactive oxygen species from reaching the bacterial cell surface and thereby reducing their effect [14].

Engineered bioactive chitosan nanoparticles have been shown in earlier investigations to retain their effective antibiofilm activity up to 90 days of aging [14] and antibacterial action even in the presence of tissue inhibitors such as dentin matrix and bacterial remnants [31]. The current study demonstrated that CSnp significantly reduced residual two and six-week-old root canal biofilms compared to conventional disinfection alone based on microbiological quantification of pulverized root sections which was consistent with the results obtained from the analysis of confocal laser scanning microscopy images. It has been emphasized that complementing fluorescent image-based microscopy with microbiological quantification method is required for precise assessment of biofilm [32]. Viability staining followed by CLSM signified the true viability of the biofilm bacteria, whereas, pulverization technique provided more reliable sampling and accurate representation of remaining cultivable bacteria in the root canal lumen and within the dentinal tubules than other sampling methods [33,34]. The apical 5 mm of roots were resected and pulverized to expose the bacterial cells as this segment can harbor bacteria in spite of chemo-mechanical debridement with canal irrigants [33]. Moreover, to ensure that sampling was contamination free, the external root surfaces were sealed throughout the incubation period of the root canal biofilm, then after intracanal treatment, their epoxy resin coats were removed, and they were disinfected and finally checked for sterility prior to pulverization. Bacterial enrichment of pulverized specimens for three hours in BHI broth increased the number of cultivable bacteria in all tested groups compared to immediate plating (data not shown), except for both groups of CSnp treated two-week-old biofilm where no bacterial growth was evident even after incubation. This emphasizes the significance of bacterial enrichment phase, as previously described [35], since the bacteria could be in a viable but nonculturable status immediately after treatment.

Six-week-old root canal biofilm showed more residual live bacteria than two-week-old biofilm, which was in accordance with previous studies in which the biofilm was reported to develop more resistance to antibacterial agents after two to three weeks of maturation [36]. Further, the complexity of the biofilm matrix presents a challenge to the host immune system [37]. Thus, six-week-old biofilm was selected for the second phase of the study to represent a more challenging model for interaction with macrophages. Engineered chitosan nanoparticles antibacterial action involves electrostatic interaction between positively-charged CSnp and negatively-charged bacterial cell which affects bacterial cell permeability leading to leakage of intracellular components and cell death [38]. 

Contrarily, eukaryotic cells such as macrophages, uptake CSnp intracellularly, mediated by endocytosis, and degrade them via lysosomal and multivesicular body pathways [39]. Macrophages are one of the major players in the host defense mechanisms against infection and their presence in human inflammatory periapical lesions has been long recognized in different phases of the disease [40]. They have the ability to shift functional phenotypic presentation in response to changes in their microenvironment. Macrophages are activated by microorganisms and their by-products such as lipoteichoic acid, lipopolysaccharide and chemical mediators [3], which promote the release of pro-inflammatory cytokines and cytotoxic molecules including TNF-α, IL-1, IL-6, IL-8 and nitric oxide [9]. An earlier study by Mathew et al. showed that *E. faecalis* (ATCC29212), that is known to produce thick biofilms with extracellular polymeric substance, could attenuate the proinflammatory response by macrophages compared with their planktonic counterpart [41]. 

In the current investigation, we examined the inflammatory response of CSnp treated biofilm using RAW264.7 murine macrophage cell line which has been widely used as an inflammatory model in-vitro. CSnp treated *E. faecalis* biofilms interacting with macrophages demonstrated significant reduction in the production of proinflammatory markers (TNF-α, IL-1β and IL-6) compared to conventional disinfection alone. The distinct drop in the level of TNF-α observed after 48 h of interaction between either untreated or conventionally treated biofilm and macrophages, could be attributed to the considerable reduction in the macrophage cell viability, as well as the instability of TNF-α which is a markedly degradable cytokine [42]. TNF-α, IL-1 and IL-6 play an important role in inducing periapical bone resorption and promoting the production of other inflammatory mediators, which in turn would magnify the disease process [43]. Thus, restraining these proinflammatory cytokines using CSnp would help modulate the periapical tissue inflammation and modify the microenvironment to be more conducive to organized healing. 

CSnp treatment restrained the production of nitric oxide robustly over the time course of interaction while the macrophage cell viability was not only maintained but it was significantly enhanced at 72 h. These results clearly demonstrated that the inhibitory effect of CSnp on proinflammatory mediators’ production may not be attributed to cytotoxic effects. On the other hand, production of TGF-β1, a well-known healing-promoting cytokine, showed progressive rise overtime in CSnp treated biofilm groups. TGF-β has been reported to inhibit T-helper 1 and T-helper 17 immune responses [43]. Further, CSnp treatment resulted in significantly the highest cells spreading area after 48 and 72 h of interaction, indicating actin polymerization and cytoskeletal rearrangement of macrophages. Both CSnp tested formulations demonstrated comparable results, with no significant difference between them in terms of antibiofilm activity and inflammatory response; moreover, they would provide additional advantages comprising restoration of the mechanical and structural stability of root canal dentin [44]. These findings warrant further investigations regarding the immunomodulatory potential of engineered bioactive chitosan nanoparticles on macrophages and other periapical tissue cells as well as their molecular mechanism of action.

## 5. Conclusions

In summary, organotypic infected root canal model has been characterized and the present study highlighted the antibiofilm efficacy of engineered bioactive CSnp for the treatment of root canal biofilm bacteria as well as altering proinflammatory response of RAW macrophages. Engineered nanoparticles treated biofilm maintained the survival of interacting macrophages up to 72 h while increasing its spreading area over time. Therefore, CSnp possess not only antibiofilm capability but also an immune modulatory effect on macrophages contrary to conventional chemicals. This could potentially modulate periapical inflammation that would promote healing in endodontic treatment. Further studies with ex-vivo or in-vivo models are required to validate its potential application in modulating periapical tissue inflammation.
